# A Bioinformatics Investigation into the Pharmacological Mechanisms of Sodium-Glucose Co-transporter 2 Inhibitors in Diabetes Mellitus and Heart Failure Based on Network Pharmacology

**DOI:** 10.1007/s10557-021-07186-y

**Published:** 2021-05-24

**Authors:** Ziling Mai, Huanqiang Li, Guanzhong Chen, Enzhao Chen, Liwei Liu, Zhubin Lun, Wenguang Lai, Chunyun Zhou, Sijia Yu, Jin Liu, Shiqun Chen, Jiyan Chen, Yong Liu

**Affiliations:** 1 Guangdong Provincial People’s Hospital, School of Biology and Biological Engineering, South China University of Technology, Guangzhou, China; 2 Department of Cardiology, Guangdong Provincial Key Laboratory of Coronary Heart Disease Prevention, Guangdong Cardiovascular Institute, Guangdong Provincial People’s Hospital, Guangdong Academy of Medical Sciences, Guangzhou, China; 3 Guangdong Provincial People’s Hospital, School of Medicine, South China University of Technology, Guangzhou, China; 4 The Second School of Clinical Medicine, Southern Medical University, Guangzhou, China; 5 The First School of Clinical Medicine, Guangdong Medical University, Zhanjiang, China

**Keywords:** Diabetes mellitus, Heart failure, Sodium-glucose co-transporter 2 inhibitors, Network pharmacology

## Abstract

**Purpose:**

Diabetes mellitus (DM) is a major risk factor for the development of heart failure (HF). Sodium-glucose co-transporter 2 (SGLT2) inhibitors have demonstrated consistent benefits in the reduction of hospitalization for HF in patients with DM. However, the pharmacological mechanism is not clear. To investigate the mechanisms of SGLT2 inhibitors in DM with HF, we performed target prediction and network analysis by a network pharmacology method.

**Methods:**

We selected targets of SGLT2 inhibitors and DM status with HF from databases and studies. The “Drug-Target” and “Drug-Target-Disease” networks were constructed using Cytoscape. Then the protein–protein interaction (PPI) was analyzed using the STRING database. Gene Ontology (GO) biological functions and Kyoto Encyclopedia of Genes and Genomes (KEGG) pathways were performed to investigate using the Bioconductor tool for analysis.

**Results:**

There were 125 effective targets between SGLT2 inhibitors and DM status with HF. Through further screening, 33 core targets were obtained, including SRC, MAPK1, NARS, MAPK3 and EGFR. It was predicted that the Rap1 signaling pathway, MAPK signaling pathway, EGFR tyrosine kinase inhibitor resistance, AGE-RAGE signaling pathway in diabetic complications and other signaling pathways were involved in the treatment of DM with HF by SGLT2 inhibitors.

**Conclusion:**

Our study elucidated the possible mechanisms of SGLT2 inhibitors from a systemic and holistic perspective based on pharmacological networks. The key targets and pathways will provide new insights for further research on the pharmacological mechanism of SGLT2 inhibitors in the treatment of DM with HF.

**Supplementary Information:**

The online version contains supplementary material available at 10.1007/s10557-021-07186-y.

## Introduction

Diabetes mellitus (DM) is a metabolic disorder characterized by hyperglycemia due to partial or complete insulin deficiency and/or insulin resistance, which affects 8.5–9.3% of the population worldwide, rising to an expected 10.2% (578 million) by 2030 and 10.9% (700 million) by 2045 [1, 2]. Heart failure (HF) is a substantial but frequently overlooked complication of DM [3]. Several studies show that the incidence of HF is 2-5 times as high in diabetic patients as in those without DM [4]. Furthermore, diabetic patients with HF have longer HF-related hospital stays, more frequent HF-related readmissions and higher risk for cardiovascular mortality than patients with HF but without DM [5–7]. However, treating patients with these concomitant diseases can be challenging. Some drugs have been recommended for the treatment of DM with HF, such as metformin and sulfonylureas, but they are insufficient in treating diabetes with heart failure. For instance, metformin alone is often not enough to keep glycemia under control, and thus does not significantly improve patients’ condition [8]. Sulfonylureas, used as second-line or third-line treatments for diabetic patients with heart failure according to the position statement of the European Society of Cardiology/Heart Failure Association, are commonly prescribed in DM but associated with weight gain and hypoglycemia, which are detrimental in heart failure [9–11]. Therefore, there is a compelling impetus to explore potential strategies to reduce the risk of HF in patients with DM.

Sodium-glucose co-transporter 2 (SGLT2) inhibitors, a class of glucose-lowering therapies, including dapagliflozin, canagliflozin, empagliflozin and ertugliflozin, have been approved by the US Food and Drug Administration for the treatment of type 2 diabetes mellitus [12]. SGLT2 inhibitors can inhibit proximal renal tubular sodium and glucose reabsorption to increase the urinary excretion of glucose, thereby reducing blood sugar [13]. Data to date suggest that this agent achieves a statistically significant and clinically relevant reduction in the risk of CV, renal outcomes and overall mortality beyond simply reducing plasma glucose levels. Clinical trials from many countries, including the EASEL Population-Based Cohort Study [14], the EMPA-REG OUTCOME trial [15] and the CVD-REAL 2 study [16], have proven that SGLT2 inhibitors reduce the risk of non-fatal myocardial infarction, non-fatal stroke, and all-cause death, as well as the risk of hospitalization and death associated with heart failure. Moreover, it can reduce the decline in renal function to protect patients from dialysis and renal failure as reported in the DARWIN-T2D and a Scandinavian cohort study [17, 18]. Most attention has focused on the pleiotropic effects of SGLT2 inhibitors on cardiac function and their potential benefits with regard to heart failure and mortality rates. Four large cardiovascular outcomes trials, the EMPA­REG OUTCOME trial [15], the CANVAS Program 30 [19], the DECLARE-TIMI 58 trial [20] and the VERTIS CV [21] trial, have demonstrated a significant and consistent reduction in heart failure events with SGLT2 inhibitors. Based on these composite data, SGLT2 inhibitors represent an important new therapeutic approach for the prevention of heart failure in at-risk patients with diabetes mellitus. And this benefit extends to patients without diabetes who have heart failure with reduced ejection fraction. The DAPA-HF study is a prospective randomized placebo-controlled trial to determine the effectiveness and safety of dapagliflozin in patients with heart failure with reduced ejection fraction based on conventional treatment. The results of the trial showed that, compared with placebo, patients with DM experienced a similar reduction in the risk of hospitalization for HF as patients without DM. This finding suggests that other actions of SGLT2 inhibitors beyond glucose-lowering might play a role in the beneficial effects of these agents in patients with HF. Therefore, the results of this large study further support the benefits of SGLT2 inhibitors in the treatment of HF [22]. However, the therapeutic targets and mechanism of SGLT2 inhibitors acting on HF have not yet been revealed, especially in patients with DM, which needs to be further explored and analyzed.

**Fig. 1:**
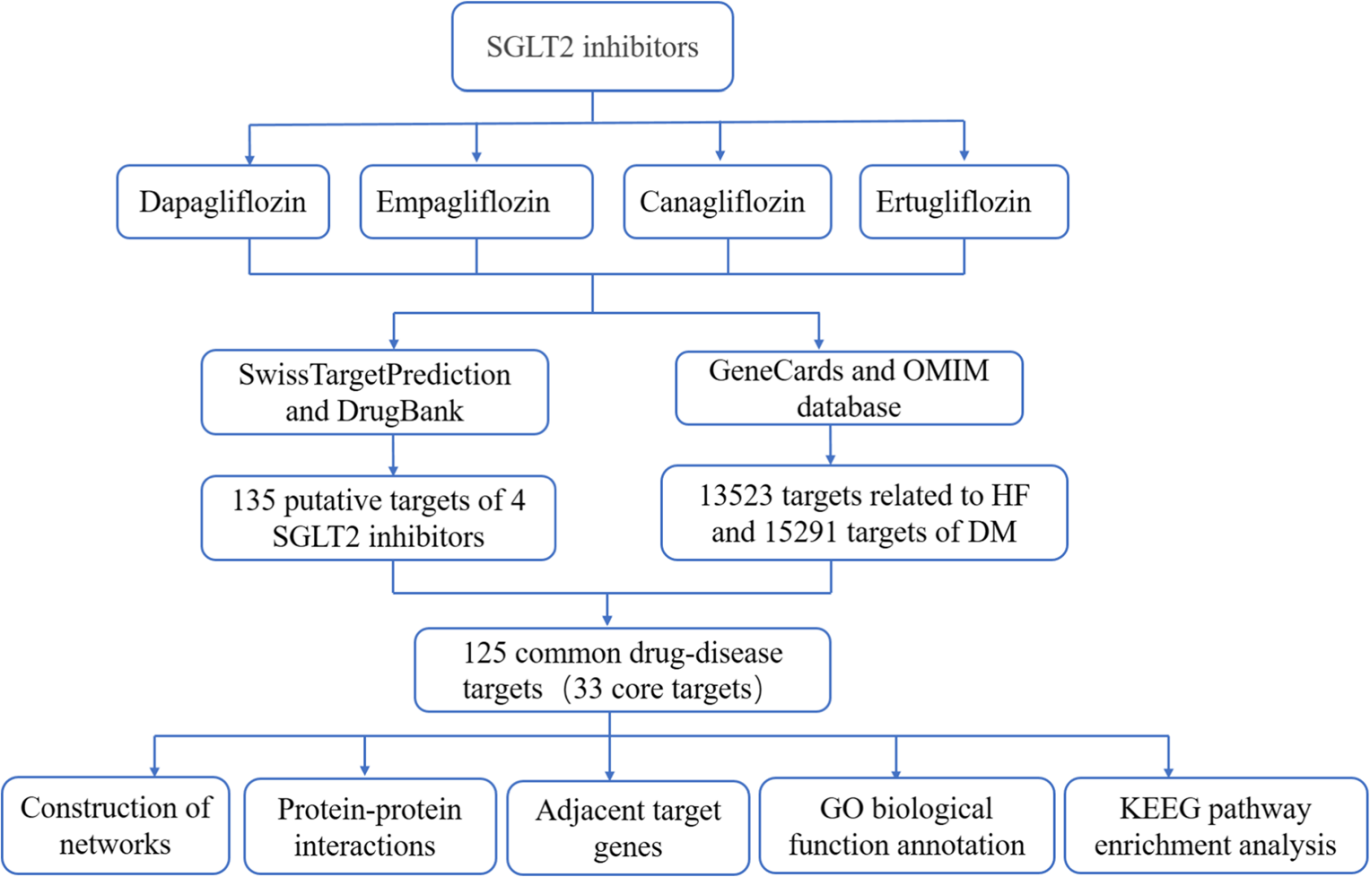
The workflow of the network pharmacology strategies for determining the pharmacological mechanisms of the SGLT2 inhibitors in diabetes with heart failure through cluster and pathway analysis.

With the development of high-throughput sequencing and computer technology, huge new bioinformatics networks have emerged. Network pharmacology, one such bioinformatics network, aims to construct a multilevel network through various database searches, high-throughput omics data analysis and computer simulations to analyze the relationship of medicines, diseases and targets [23]. Compared with traditional experimental pharmacology methods, network pharmacology is based on a comprehensive system which is more efficient for exploring the target and pathway relationships between drugs and diseases. Therefore, we applied network pharmacology analysis to systematically excavate the action targets of SGLT2 inhibitors in DM with HF to analyze the biological pathways involved, which will lay a good foundation for further in-depth exploration of the mechanism of SGLT2 inhibitors acting on DM with HF. The study flowchart of this network pharmacology-based study of SGLT2 inhibitors is shown in Fig. 1.

## Methods

### Prediction of SGLT2 Inhibitor-Related Targets

The chemical structures of four SGLT2 inhibitors, namely canagliflozin, dapagliflozin, empagliflozin, and ertugliflozin, were obtained using Pubchem (https://pubchem.ncbi.nlm.nih.gov/), which is an open chemistry database with 96,502,248 compositions, of which 3,151,393 have been tested. Then SwissTargetPrediction (http://www.swisstargetprediction.ch/), a tool for target prediction according to two-dimensional and three-dimensional similarity measures with known ligands, was selected to predict potential targets for four SGLT2 inhibitors by putting their chemical structures into this platform [24]. Additionally, SGLT2 inhibitor-related genes were collected from DrugBank (https://www.drugbank.ca/), which is a unique bioinformatics and chemical informatics database, containing 11,628 drugs and related chemical information, drug targets, protein data, and so on [25]. With further correction and transformation by the retrieval of Universal Protein Resource (UniProt, http://www.uniprot.org/), all the SGLT2 inhibitor-related genes were normalized into consistent symbols for subsequent analysis.

### The Prediction of Known Therapeutic Targets in DM Status with HF

With the keywords of “heart failure”, “diabetes”, and “diabetes mellitus”, target genes related to HF and DM were found in the GeneCards (https://www.genecards.org) and OMIM (https://www.omim.org) databases. The GeneCards database includes more than 7000 human genes, and each gene has an approved gene symbol. The OMIM database is a knowledge base of human genes and hereditary diseases. The two methods are a good reference for the collection of disease targets. Meanwhile, we collected disease targets from the studies reporting existing network analysis on actual patients with DM status with HF [26, 27]. According to the targets of SGLT2 inhibitors and diseases, the repeated targets of the two were screened by Excel, and their intersection targets were obtained. According to their intersection, we obtained a Venn diagram on the Venny website (https://bioinfogp.cnb.csic.es/tools/venny/).

### Construction of the Network Model

The four SGLT2 inhibitors, their corresponding targets and the intersection targets of diseases and drugs were sorted and input into Cytoscape v3.6.1 to construct the following networks: (1) network between four SGLT2 inhibitors (canagliflozin, dapagliflozin, empagliflozin, and ertugliflozin) and their corresponding targets; (2) network between four SGLT2 inhibitors, intersection targets, and diseases (DM status with HF). Cytoscape is a software program that can efficiently express the interaction between protein and protein, protein and DNA or gene, in order to visualize network relationships.

### Screening of Core Targets of SGLT2 Inhibitors in Treatment of DM with HF

The STRING (https://string-db.org) dataset is now one of the largest PPI datasets, including co-expression data, biomedical literature data, high-throughput data and genomic background data. In the platform, “Multiple proteins” was selected and the organism was selected as “*Homo sapiens*.” The intersection targets of SGLT2 inhibitors and DM status with HF were then imported to construct the protein–protein interaction network. In order to ensure the high confidence of information, the scoring condition was set to > 0.90, and the isolated proteins in the figure were hidden. The file was exported in “TSV” format, and then Cytoscape v3.6.1 was used to analyze the topological properties of the PPI network. To detect the core targets in the common targets between drugs and diseases, the degrees of targets were calculated using the Cytohubba plugin based on Cytoscape.

### Construction of Protein–Protein Interaction (PPI) Network of Core Targets

The STRING online platform was used to build the PPI network of core targets. After selecting the multiple proteins module, the Gene Name list of target proteins was uploaded to the network, limiting the species to *Homo sapiens*, and the confidence score was set to > 0.9. In the PPI diagram, each solid circle represents a gene, and the middle of the circle shows the structure of the protein, while the circles are connected by lines of different colors. Each line represents the biological process between protein and protein, including regulation of gene expression, signal transduction, cell migration and so on.

### GO Enrichment Analysis and KEGG Pathway Enrichment Analysis

In order to better understand the potential biological process of core genes, KEGG (Kyoto Encyclopedia of Genes and Genomes) and GO (Gene Ontology) were analyzed for pathway functional enrichment using the clusterProfiler software package on the R platform. The interaction network was constructed using the Top-Go package of the R platform.

## Results

### Screening Potential Related Targets of SGLT2 Inhibitors

**Fig. 2:**
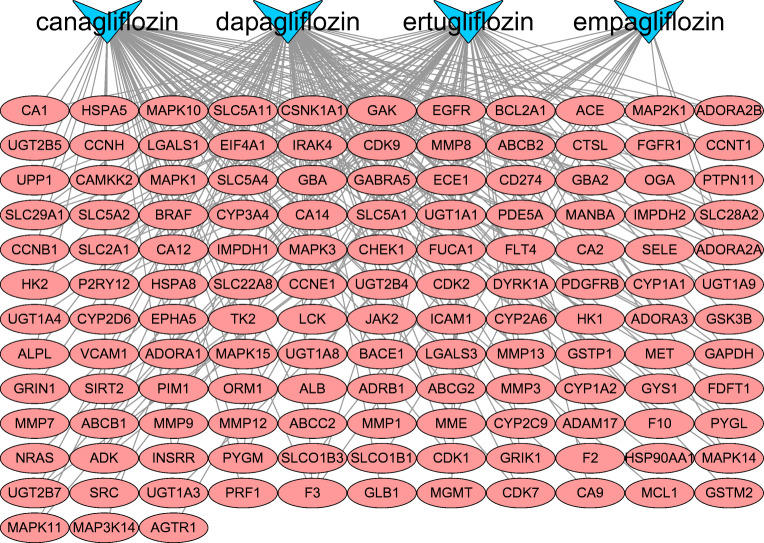
Interaction network to indicate drug-target composited of 4 SGLT2 inhibitors (blue) and 136 targets (pink).

The PubChem platform was used to acquire the molecular structure of dapagliflozin empagliflozin, canagliflozin and ertugliflozin. The details of their structure are shown in Table 1. According to their molecular structure, the four SGLT2 inhibitors have a common parent nucleus, although their side-chain substituents are somewhat different. After importing their structures into the SwissTargetPrediction database for target matching and prediction, we screened the 213 targets with probability > 0, of which 71 targets were in dapagliflozin, 65 in canagliflozin, 61 in ertugliflozin and 16 in empagliflozin. We also accessed the DrugBank database to find 37 targets on the four SGLT2 inhibitors, with 11 targets in dapagliflozin, 10 in empagliflozin, 8 in canagliflozin and 8 in ertugliflozin. By integrating and eliminating duplicate targets in the two databases, a total of 135 targets with potential effects of SGLT2 inhibitors were obtained. Then, introducing them into Cytoscape v3.6.1 software for analysis, we constructed a visualized drug-target network (Fig. 2). There are 139 nodes (135 targets, 4 drugs) and 252 edges shown in Fig. 2, where the pink nodes represent SGLT2 inhibitors, the blue nodes represent the drug targets (the predicted target) and the edges represent the interactions between the drugs and the targets. This reflects the potential mechanism of interaction between SGLT2 inhibitors and multiple targets.

**Table 1:**
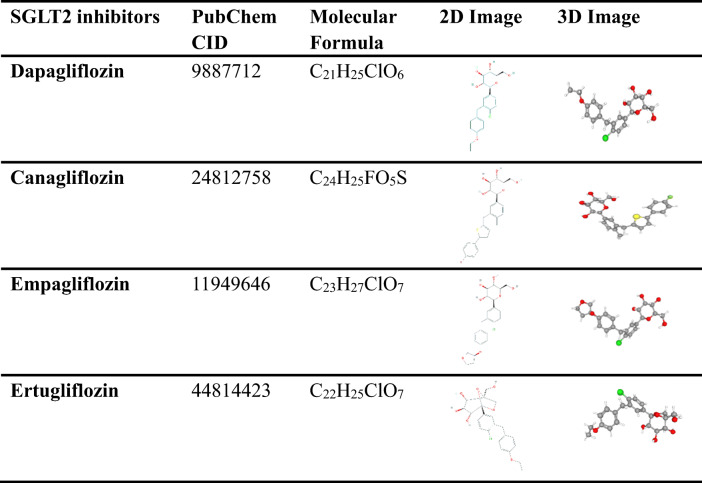
Information on four SGLT2 inhibitors from PubChem

### Construction and Analysis of Drug-Target-Disease Network

**Fig. 3:**
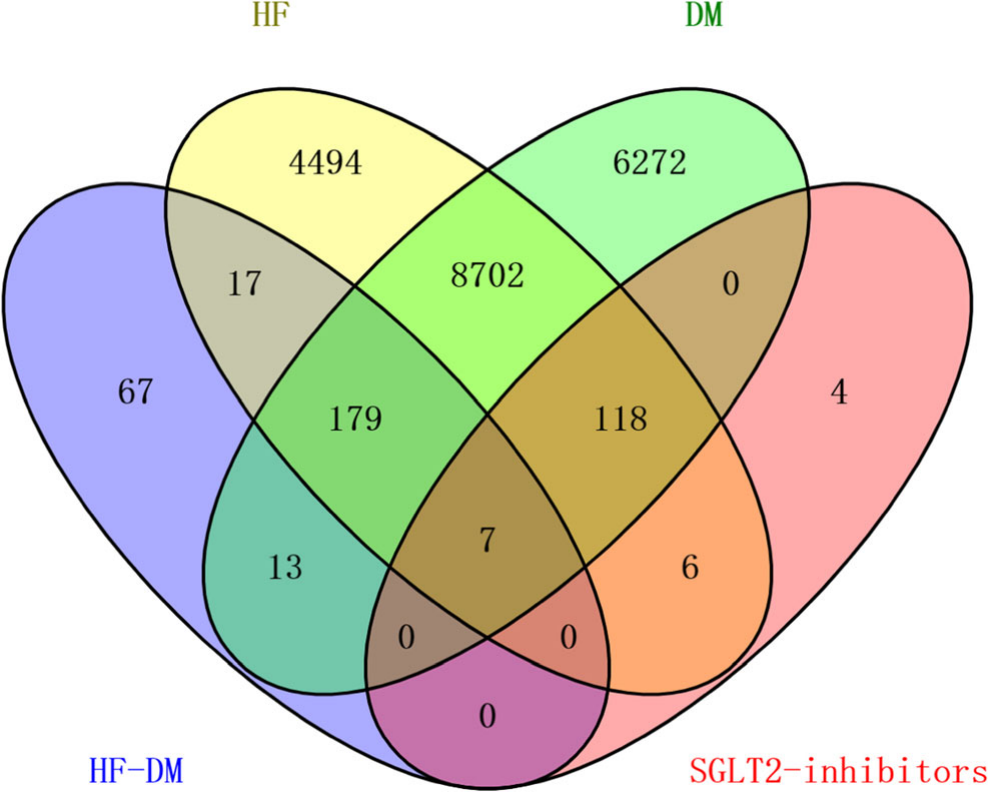
The numbers of SGLT2 inhibitors and diabetes status with heart failure-related targets are shown in the Venn diagram. There were 13,523 targets of HF from the databases (upper left), 15,291 targets of DM from the database (upper right), 283 targets of HF-DM from studies (bottom left), 135 targets of SGLT2 inhibitors (bottom right) and 125 common targets between SGLT2 inhibitors and diabetes status with heart failure.

**Fig. 4:**
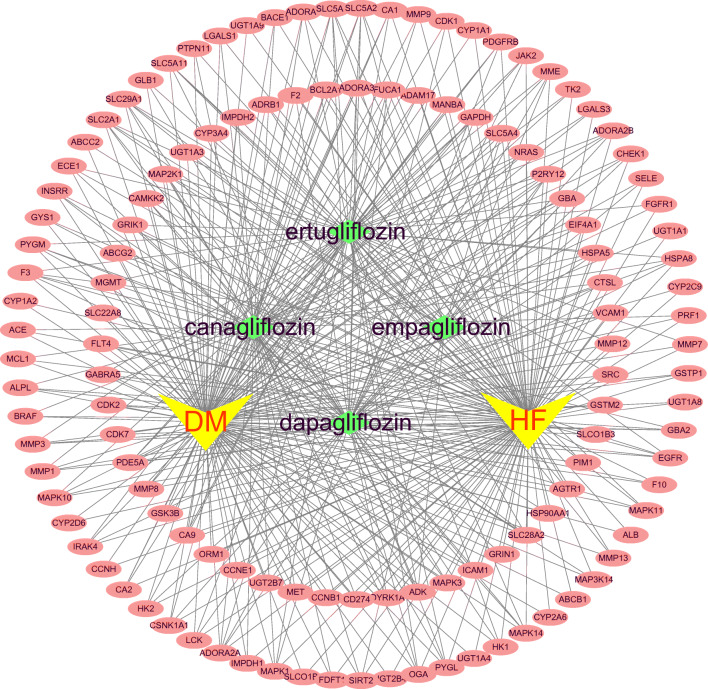
The drug-target-disease networks of SGLT2 inhibitors and diabetes status with heart failure. The yellow nodes represent DM and HF; the green nodes represent four SGLT2 inhibitors; the pink nodes represent 125 common targets.

After repetition was removed, a total of 13,523 targets related to HF and 15,291 targets corresponding to DM were collected from the GeneCards and the OMIM databases. In addition, 283 targets in the context of existing network analysis on actual patients with DM with HF were also obtained. Among these, 125 common targets were shared among potential targets of SGLT2 inhibitors, known HF-related targets and DM-related targets by an online Venn diagram-drawing platform, seven of which came from actual patients with DM with HF in the studies (Fig. 3), In addition, 125 “drug-disease” common targets were introduced into Cytoscape v3.6.1 software to construct a visualized drug-target-disease network (Fig. 4). A total of 131 nodes (125 targets, 4 drugs, 2 diseases) and 441 lines are shown in the network. The purple nodes are the four SGLT2 inhibitors (dapagliflozin, empagliflozin, canagliflozin and ertugliflozin); the red nodes are HF and DM; the green nodes represent 125 common targets. In this network, the average target number of each SGLT2 inhibitor is 6.73. Thus, there is an interaction between one SGLT2 inhibitor and multiple targets in the treatment of DM with HF. With regard to the targets, the top three in degree are SLC5A2, SLC5A1, and SLC2A1, well known as the sodium-dependent glucose co-transporter gene, which can interact with the four SGLT2 inhibitors, respectively. These targets connect the relationship between SGLT2 inhibitors and disease, which provides a better reference for exploring the mechanism of SGLT2 inhibitors in the treatment of DM with HF.

### Core Targets of SGLT2 Inhibitors in DM Status with HF

**Fig. 5:**
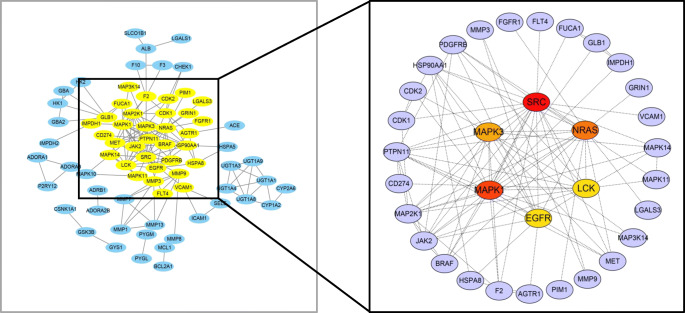
The process of screening core targets by Cytohubba according to degree value. From 125 common targets (left), 33 core targets (right) ranked in the front of the degree value were selected.

A total of 125 common targets were imported into the STRING online platform, and a file in “TSV” format was exported. It was then imported into Cytoscape v3.6.1, and the function "Cytohubba" was used to calculate the degree of targets to find the core targets. Thirty-three core targets of SGLT2 inhibitors in the treatment of DM with HF were screened (Fig. 5), among which red, orange, yellow and purple nodes represent a gradual decrease in the degree value from large to small. The specific information for the core targets is shown in Table 2.

**Table 2:** Specific information for core targets

Number	Uniprot ID	Gene name	Protein name	Counts
1	P12931	SRC	Tyrosine-protein kinase Lck	19
2	P28482	MAPK1	Mitogen-activated protein kinase 1	17
3	P01111	NRAS	GTPase NRas	16
4	P27361	MAPK3	Mitogen-activated protein kinase 3	15
5	P00533	EGFR	Epidermal growth factor receptor	12
6	P06239	LCK	Tyrosine-protein kinase Lck	12
7	Q06124	PTPN11	Tyrosine-protein phosphatase nonreceptor type 11	11
8	Q02750	MAP2K1	Dual specificity mitogen-activated protein kinase kinase 1	11
9	O60674	JAK2	Tyrosine-protein kinase JAK2	10
10	P07900	HSP90AA1	Heat shock protein HSP 90-alpha	7
11	P09619	PDGFRB	Platelet-derived growth factor receptor beta	6
12	P15056	BRAF	Serine/threonine-protein kinase B-raf	6
13	Q16539	MAPK14	Mitogen-activated protein kinase 14	5
14	P00734	F2	Prothrombin	5
15	P08581	MET	Hepatocyte growth factor receptor	5
16	P06493	CDK1	Cyclin-dependent kinase 1	4
17	P24941	CDK2	Cyclin-dependent kinase 2	3
18	P30556	AGTR1	Type-1 angiotensin II receptor	3
19	P11142	HSPA8	Heat shock cognate 71 kDa protein	3
20	Q15759	MAPK11	Mitogen-activated protein kinase 11	3
21	P04066	FUCA1	Tissue alpha-L-fucosidase	3
22	P16278	GLB1	Beta-galactosidase	3
23	P20839	IMPDH1	Inosine-5'-monophosphate dehydrogenase 1	3
24	Q9NZQ7	CD274	Programmed cell death 1 ligand 1	2
25	P14780	MMP9	Matrix metalloproteinase-9	2
26	P08254	MMP3	Stromelysin-1	2
27	Q99558	MAP3K14	Mitogen-activated protein kinase kinase kinase 14	2
28	Q05586	GRIN1	Glutamate receptor ionotropic, NMDA 1	1
29	P19320	VCAM1	Vascular cell adhesion protein 1	1
30	P11309	PIM1	Serine/threonine-protein kinase pim-1	1
31	P17931	LGALS3	Galectin-3	1
32	P11362	FGFR1	Fibroblast growth factor receptor 1	1
33	P35916	FLT4	Vascular endothelial growth factor receptor 3	1

### Analysis of 33 Core Targets of the Protein–Protein Interaction Network (PPI)

**Fig. 6:**
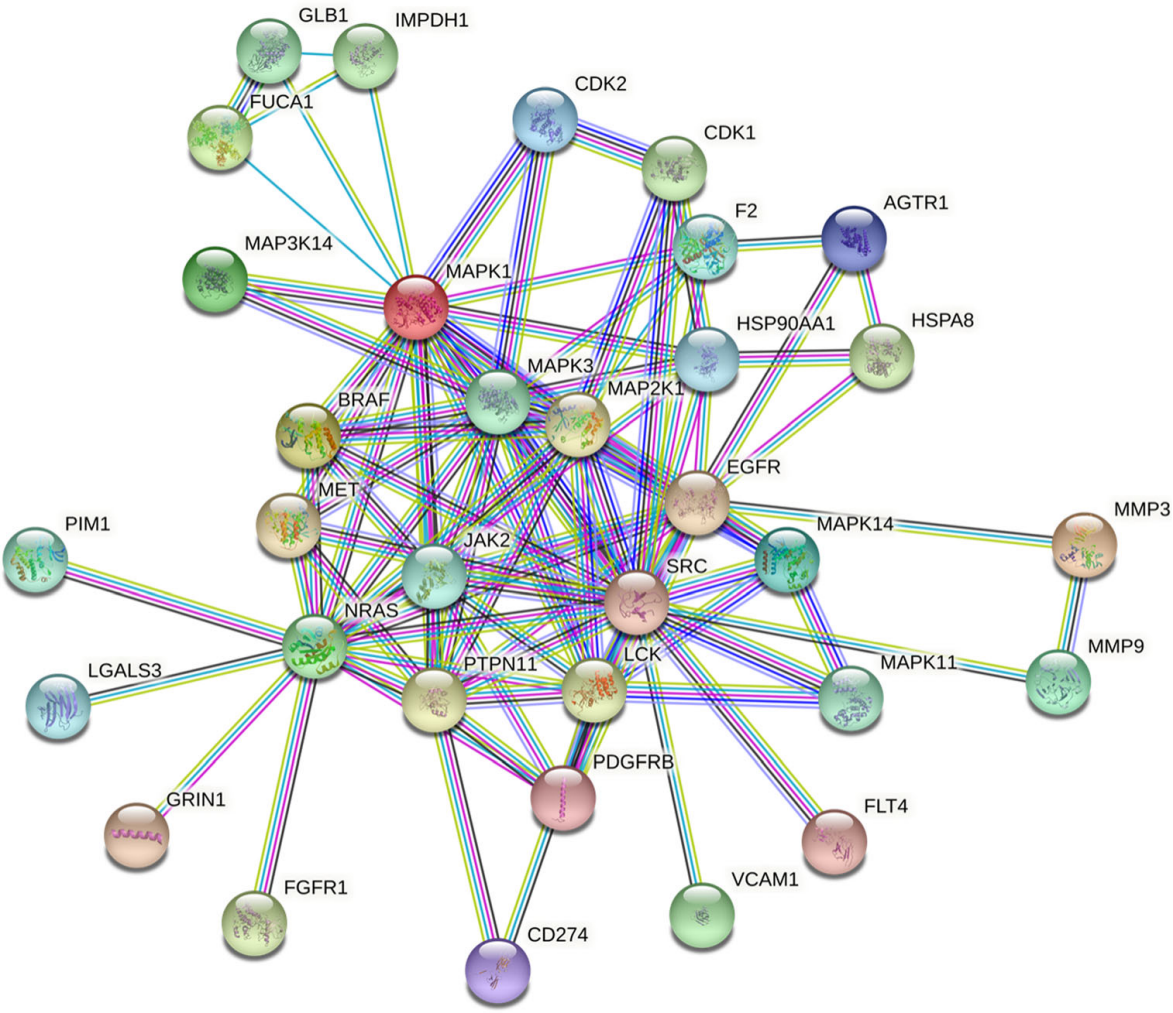
The protein–protein interaction (PPI) network based on 33 core targets of SGLT2 inhibitors in diabetes status with heart failure. Network nodes represent different proteins. The structures in the nodes are the protein structures. Edges represent protein–protein associations, and the line thickness indicates the strength of data support.

**Fig. 7:**
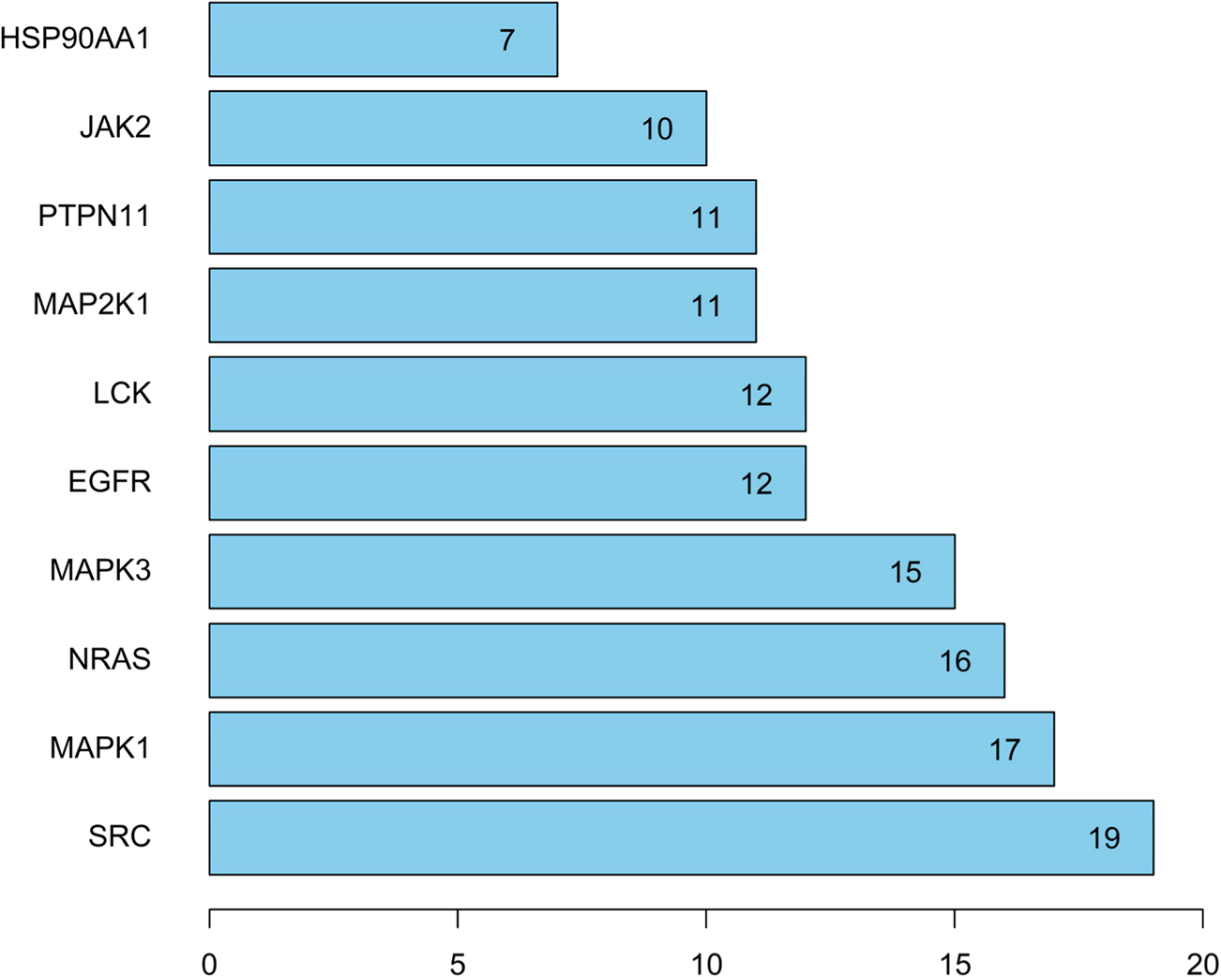
Protein interaction relationship histogram of SGLT2 inhibitors.

The STRING database platform was used to construct a network of target protein interactions, and the 33 core targets of SGLT2 inhibitors-HF-DM were imported. By selecting the species “*Homo sapiens*” and setting a combined score > 0.9 threshold, the final protein–protein interaction network was obtained (Fig. 6). As can be seen from Fig. 6, there are a total of 33 solid circles of many colors, each circle representing a key target gene, and the center of the dot shows the protein structure of the target gene. The statistical analysis was performed on each target gene to obtain the top targets with the number of adjacent genes  ≥ 10 (shown in Fig. 7), revealing that SRC, MAPK1, NARS, MAPK3 and EGFR are the top five hub targets, which may be the key targets for SGLT2 inhibitors in the treatment of DM with HF.

### GO Biological Function Annotation and KEGG Pathway Enrichment Analysis for Targets

**Fig. 8:**
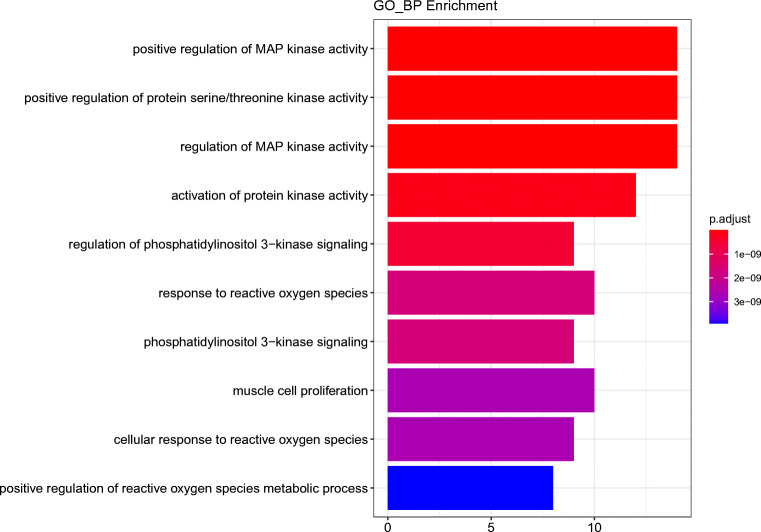
Enrichment analysis of Gene Ontology (GO) biological process of 33 core genes related to SGLT2 inhibitors on diabetes status with heart failure.

To study the mechanism of SGLT2 inhibitors in DM with HF more systematically, the 33 core target genes of the SGLT2 inhibitors-HF-DM intersection were introduced into R statistical programming language to analyze the GO biological function and KEGG signaling pathway. Filtering with P value <0.05 and Q value <0.05 as threshold values, we selected the 10 biological processes of 33 core targets, as shown in Fig. 8. The results indicate that the targets are mainly associated with positive regulation of MAP kinase activity, positive regulation of protein serine/threonine kinase activity, regulation of MAP kinase activity, activation of protein kinase activity, regulation of phosphatidylinositol 3-kinase signaling, response to reactive oxygen species, phosphatidylinositol 3-kinase signaling, muscle cell proliferation, cellular response to reactive oxygen species and positive regulation of reactive oxygen species metabolic processes.

**Fig. 9:**
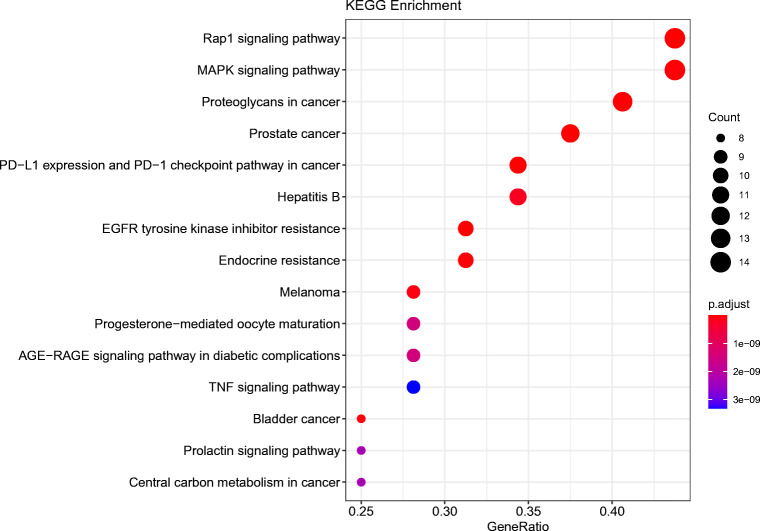
Kyoto Encyclopedia of Genes and Genomes (KEGG) pathway for 33 core targets of SGLT2 inhibitors on diabetes status with heart failure.

Based on the KEGG enrichment analysis of 33 key targets, we found the top 15 signal paths with high confidence (P value<0.05) selected for analysis in Fig. 9. According to the results, the core targets might affect signaling pathways including the Rap1 signaling pathway, MAPK signaling pathway, EGFR tyrosine kinase inhibitor resistance and AGE-RAGE signaling pathway in diabetic complications, which predicts that SGLT2 inhibitors might be effective for treatment of DM with HF by regulating the aforementioned signaling pathways.

**Fig. 10:**
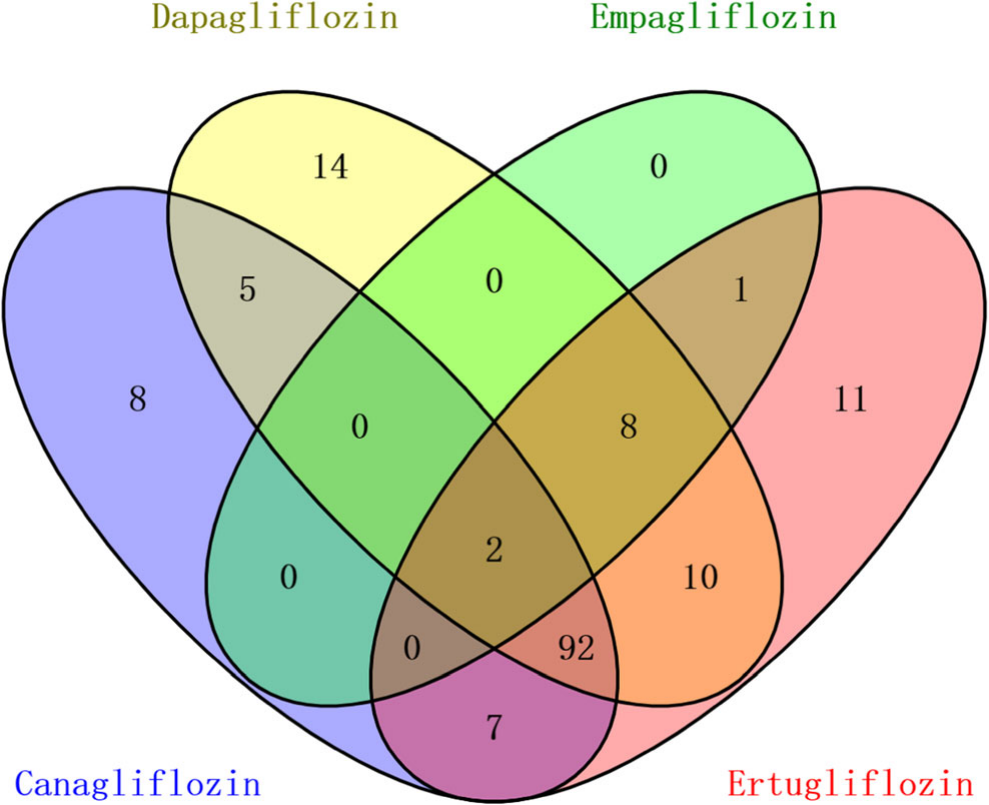
The KEGG pathways of the four drugs were shown in the Venn diagram.

In addition, we performed KEGG analysis on the targets of the four SGLT2 inhibitors to find their pathways separately. We intersected the pathways of the four drugs in a Venn diagram to explore the same and different signaling pathways among them (Fig. 10). We identified 387 pathways in total across four drugs. Although 33 pathways were present in a certain SGLT2 inhibitor, a total of 125 pathways were overlapped in two or more SGLT2 inhibitors, and these overlapping pathways included the four key pathways analyzed in the previous KEGG analysis results, that is, Rap1 signaling pathway, MAPK signaling pathway, EGFR tyrosine kinase inhibitor resistance and AGE-RAGE signaling pathway in diabetic complications, which demonstrates that the four pathways play an important role in the treatment of SGLT2 inhibitors in DM with HF.

## Discussion

Heart failure (HF) is a major complication of diabetes mellitus (DM). Therefore, the prevention of HF in patients with DM is critical. In clinical studies, SGLT2 inhibitors were found to be effective in treating HF among patients with DM, which aroused great interest and attention. However, the mechanism is not clear. Based on network pharmacological analysis, this study explored the mechanism of SGLT2 inhibitors in the treatment of DM with HF. In the present study, four SGLT2 inhibitors (canagliflozin, dapagliflozin, empagliflozin, and ertugliflozin) were obtained to analyze the effects on DM with HF based on the network pharmacology analysis. According to the drug-disease target network and PPI network, 33 core genes of the SGLT2 inhibitors acting on DM with HF were obtained from 125 common genes. Among the 33 core genes, the top five hub targets SRC, MAPK1, NRAS, MAPK3 and EGFR were screened according to the degree. The module analysis confirmed that SGLT2 inhibitors have the potential to influence a variety of biological pathways that play an important role in the pathogenesis of DM with HF. The key pathways were screened by KEGG analysis, mainly involving the Rap1 signaling pathway, MAPK signaling pathway, EGFR tyrosine kinase inhibitor resistance and AGE-RAGE signaling pathway in diabetic complications.

### Further Analysis of the Identified Genes

Among the top five core genes, the first, SRC, is a non-receptor tyrosine kinase that plays a role in numerous biological processes including cell adhesion, cell cycle and cell migration. It was demonstrated that SRC kinases could be activated by Ang II, which plays an important role in Ang II-mediated processes [28], including the pathophysiology of cardiac hypertrophy and remodeling, vascular thickening and heart failure [29]. Pandey et al. found that SRC activation contributed to the alteration of non-myofibrillar tension, which would impact the baseline tension in fibrotic hearts after MI or in dilated cardiomyopathy [30]. Inhibition of SRC(c-Src) activation was found to decrease endogenous ROS production and increase ATP production in diabetic mice with hyperlipidemia [31]. Safari-Alighiarloo et al. also identified SRC as a key gene for type 1 diabetes through analysis of gene expression profiles [32]. SRC may be a potential target for treatment of DM with HF disease.

The next two hub genes, MAPK1 (mitogen-activated protein kinase 1) and MAPK3 (mitogen-activated protein kinase 3), also known as ERK1 and ERK2, respectively, are members of the MAP kinase (MAPK) family. MAPK acts as an integration point for multiple biochemical signals and is involved in a wide variety of cellular processes including proliferation, differentiation, transcription regulation and development. As one of the MAPK family members, the activation of ERK1/2 has also been known to be involved in cardiac hypertrophy and dysfunction [33]. In addition, dysregulated ERK 1/2 levels were associated with dysregulation in a type 1 diabetes mouse model, which showed that ERK 1/2 can affect diabetes [34]. This suggests that MAPK1 and MAPK3 are potential targets in DM with HF.

The next gene is NRAS, an N-ras oncogene encoding a membrane protein that shuttles between the Golgi apparatus and the plasma membrane, and works in various differentiation processes and signal transduction involving the regulation of cell survival and growth, T cell activation and apoptosis. Katoh et al. reported that NRAS was one of the representative targets on cardio-miR-214 that were upregulated in human heart failure, showing that NRAS might be associated with the progression of heart failure [35]. It was also observed that hispidulin modulated multiple Pim1-interacted proteins such as NRAS and RAB18 to regulate the development of diabetic nephropathy [36]. Thus, NRAS has the potential to be a target of DM with HF.

The last of the top five hub genes is EGFR, epidermal growth factor receptor, a cell surface protein binding to epidermal growth factor that regulates cell growth, proliferation and survival, and is involved in blood pressure regulation, neointimal hyperplasia, atherogenesis and reactive oxygen. Xu et al. revealed that the enhanced myogenic constriction of the mesenteric artery in heart failure might be related to the loss of plasmalemmal caveolae in mesenteric vascular smooth muscle cells, and the increased activity of the EGFR and AT_1_ receptors was considered to be one of the mechanisms leading to this result [37]. Belmadani et al. found that elevated EGFR phosphorylation contributed to resistance artery dysfunction in type 2 diabetes [38]. Zhang et al. concluded that inhibiting EGFR slowed the progression of diabetic nephropathy by decreasing endoplasmic reticulum stress and increasing autophagy [39]. These findings demonstrate that EGFR may play an irreplaceable role in DM with HF. Overall, the top five core genes of the present study based on network pharmacology are supported by previous studies.

Moreover, the targets of our findings have some overlap with those in existing network analysis on actual patients with DM with HF, that is ALB, FDFT1, SLC22A8, ABCG2, MME, LGALS3 and FUCA1, which act as biomarkers in HF patients with DM [26, 27]. The results suggest that these targets may play an important role in the prevention and treatment of SGLT2 inhibitors in DM combined with heart failure.

### Enrichment Analysis Based on Core Targets

GO functional enrichment and KEGG pathway analysis have illustrated the role of the SGLT2 inhibitors in the gene function and signaling pathway. According to the biological processes that mainly reflect the GO functional enrichment, these core targets are focused on kinase activity, primarily in positive regulation of MAP kinase activity, positive regulation of protein serine/threonine kinase activity and so on. Among them, positive regulation of protein serine/threonine kinase activity is a core biological process that may affect the function of cGMP in the regulation of the physiological response of the natriuretic peptide (NP) system. This is because cGMP-dependent protein kinases (cGK) are exactly serine/threonine kinases widely distributed in eukaryotes [40]. The physiological response of the NP system is mainly achieved by binding to NPR-A, activating guanosine cyclase and producing cGMP [41], and the NP system has been extensively associated with the development and progression of HF [42]. It seems that SGLT2 inhibitors can affect the NP system to act on the development of HF through the biological process involving positive regulation of protein serine/threonine kinase activity.

In the enrichment of the KEGG pathway, the Rap1 signaling pathway, MAPK signaling pathway, EGFR tyrosine kinase inhibitor resistance and AGE-RAGE signaling pathway in diabetic complications were found to be the significant pathways.

The Rap1 signaling pathway is implicated in a wide range of biological processes from cell proliferation and differentiation to cell adhesion. Rap1 has been proven to play a part in the regulation of integrin affinity, adhesion, and migration in postnatal neovascularization, mainly mediating the angiogenesis pathway and serving in an important role in cardiac hypertrophy. Furthermore, Rap1B can prevent excessive vascular leakage in early diabetes mellitus by inhibiting VEGF signal transduction. By controlling telomere length, Rap1 can decrease the occurrence and development of diabetes-related cardiovascular disease [43]. Downregulation of Rap1B to reduce VEGF signal transduction can impede excessive vascular leakage in early diabetes mellitus [44]. These imply that the Rap1 signaling pathway may be involved in DM with HF.

Consistent with the core target results above, the MAPK pathway is also predicted to play a role in DM with HF. It was demonstrated that the MAPK signaling pathway cascade is initiated in cardiomyocytes through activation of G protein-coupled receptors, receptor tyrosine kinases and stress stimulation. Zhang et al. reported that the MAPK signaling pathway was found to regulate cardiomyocyte apoptosis in mice with heart failure after MI, indicating that this pathway has a potential role in heart failure disease [45]. During the process of insulin resistance, there exists a chronic inflammatory response involving the MAPK pathway in the inflammatory response of type 2 diabetes, such as in diabetic nephropathy and liver disease [46, 47].

EGFR tyrosine kinase inhibitor resistance acts on EGFR tyrosine kinase, also identical to the core genes predicted based on the PPI. Previous studies had indicated that inhibition of EGFR activity protected against progressive DN in T1 DM and T2 DM [48]. Zeng et al. concluded that EGFR inhibition reduced ROS production in the left ventricle and blunted hypertensive myocardial hypertrophy in spontaneously hypertensive rats [49]. Therefore, the EGFR tyrosine kinase inhibitor resistance may have a common effect to accelerate the progression of DM with HF.

The AGE-RAGE signaling pathway is a well-studied cascade in DM. It is found that the AGE-RAGE signaling pathway can directly mediate vascular calcification in diabetes. Additionally, the pathway can also impact diabetic complications, as it leads to oxidative stress, increased inflammation, and enhanced extracellular matrix accumulation resulting in diastolic and systolic dysfunction [50]. Fukami et al. proved that activation of the AGE-RAGE signaling pathway in diabetic complications could cause excessive production of advanced glycation end products to damage cardiomyocytes, leading to HF [51], which indicates that the AGE-RAGE signaling pathway in diabetic complications might mediate the progression of heart failure, similar to results of pathway analysis in another study [52]. Therefore, our results suggest that the four signaling pathways may be involved in the mechanisms of SGLT2 inhibitors affecting DM status with HF.

Meanwhile, other KEGG analysis results that explore the same and different signaling pathways between four SGLT2 inhibitors showed that the four key pathways mentioned above were contained in 125 pathways overlapping in two or more SGLT2 inhibitors, which also indicated that the four pathways might play a key part in the treatment of DM with HF by SGLT2 inhibitors. In addition, according to the same and different mechanistic pathways of four SGLT2 inhibitors, we suggest that even though the four SGLT2 inhibitors have a few different pathways, a large part of their pathways of action are similar. The four SGLT2 inhibitors have a common parent nucleus, although their side-chain substituents are somewhat different, leading to small differences in the efficacy of drugs [13]. Considering these factors, we think that the four SGLT2 inhibitors may have class effects on the whole, but do not rule out that they each have unique effects in some diseases. Some studies have also indicated that SGLT2 inhibitors had more internally and externally consistent class effects on HF risk reduction than the MACE composite outcome [53, 54].

Network pharmacology is indeed a new method for studying the relationship between drugs and diseases. In our study, network pharmacology revealed the potential targets of SGLT2 inhibitors, as well as targets of DM with HF, and bioinformatics was used to study the main enrichment pathways. Based on the network pharmacology, our study predicted the potential therapeutic targets of SGLT2 inhibitors in DM with HF and revealed their action on the main pathways through core genes, which explained the mechanisms of SGLT2 inhibitors in DM with HF and provided scientific evidence for SGLT2 inhibitors to treat DM with HF. However, the main limitation of this study is the lack of experimental verification. Consequently, pharmacological studies will be critical to further elucidate the relationship of SGLT2 inhibitors and DM with HF. In addition, validation of the molecular levels of our findings is necessary for the future.

## Conclusions

Taken together, our study systematically predicted, screened and analyzed the targets and pathways that might play a vital role in the biological process, which elaborated the possible mechanisms of SGLT2 inhibitors in DM status with HF. Most importantly, these results provide evidence and new insights for further research on the pharmacological mechanism of SGLT2 inhibitors.

## Electronic Supplementary information

Supplementary Table S1 The predicted targets of four SGLT2 inhibitors from SwissTargetPrediction. (CSV 35 kb)

Supplementary Table S2 The predicted targets of four SGLT2 inhibitors from DrugBank. (CSV 2 kb)

Supplementary Table S3 The related targets of heart failure from GeneCards. (CSV 1692 kb)

Supplementary Table S4. The related targets of heart failure from OMIM. (CSV 49 kb)

Supplementary Table S5. The related targets of diabetes mellitus from GeneCards. (CSV 1880 kb)

Supplementary Table S6 The related targets of diabetes mellitus from OMIM. (CSV 69 kb)

Supplementary Table S7 The detailed results of GO biological process analysis. (CSV 317 kb)

Supplementary Table S8 The detailed results of KEGG analysis. (CSV 26 kb)

Supplementary Table S9 The related targets of HF-DM from the network analysis studies conducted on patients with DM status with HF. (XLSX 13 kb)

Supplementary Table S10 The detailed results of KEGG analysis on canagliflozin. (XLSX 21 kb)

Supplementary Table S11 The detailed results of KEGG analysis on dapagliflozin. (XLSX 23 kb)

Supplementary Table S12 The detailed results of KEGG analysis on empagliflozin. (XLSX 11 kb)

Supplementary Table S13 The detailed results of KEGG analysis on ertugliflozin. (XLSX 23 kb)

## Data Availability

Author can confirm that all relevant data are included in the article and/or its supplementary information files.
